# Consequences of the accessibility of the mountain national parks in Poland

**DOI:** 10.1007/s11356-022-24197-w

**Published:** 2022-11-16

**Authors:** Sylwia Adach, Małgorzata Wojtkowska, Paweł Religa

**Affiliations:** 1 Faculty of Building Services, Hydro and Environmental Engineering, Warsaw University of Technology, Nowowiejska 20, 00-653 Warsaw, Poland; 2 Department of Processes and Products Eco-Engineering, Kazimierz Pulaski University of Technology and Humanities in Radom, Chrobrego 27, 26-600 Radom, Poland

**Keywords:** Mountain landscape, Waste, Devastation, National park, Tourist, Environmentally valuable area

## Abstract

In Poland, mountain national parks are visited by about 8 million tourists annually. As a result, national parks must have a properly developed infrastructure to accommodate such a large number of people. Tourism development in mountain national parks satisfies the needs of tourist participants and should increase the attractiveness of the area, fit into the cultural concepts of a given region, and promote its development. The research aims to determine the consequences of making mountain national parks available in Poland and determine the factors determining the attractiveness of the protected area and the related tourist burden. Nine mountain national parks located in Poland were selected for the research. An interview was conducted with employees of mountain national parks concerning (a) the tourist burden on the national park, (b) the tourist management of the national park and its surroundings, and (c) the impact of tourist traffic on the natural environment. Parameters characterizing the intensity and spatial character of tourist traffic, tourist management, and the influence of tourist traffic on the natural environment of mountain national parks are discussed. The study presents the parameters determining: tourist traffic density, tourist route density, and tourist traffic intensity. A map of the development of the surroundings of national parks was presented, and its influence on tourist traffic was determined. The obtained results were discussed in the context of the consequences of tourist traffic in mountain national parks. The result of the work was the development of a map of the tourist load of mountain national parks in Poland. Tourism is of crucial importance for the proper functioning of national parks. However, if not properly cultivated, it threatens the fauna and flora of such areas. Rational use of natural resources by tourists visiting mountain national parks is crucial to maintain the balance between man and nature. Proper supervision and management in the national park, as well as the collective responsibility of tourists visiting national parks and the community living in their vicinity, is of fundamental importance for the proper functioning of the system in national parks and nearby tourist destinations.

## Introduction

The national park area is a property with extraordinary, often unique natural values. By definition, it is an area that has been preserved in its natural or close to a natural state and is under legal protection. National parks protect natural resources and processes of outstanding value, where nature is relatively best preserved. They aim to preserve biological diversity, resources, creations and components of inanimate nature, and landscape and animate nature. The national park area is primarily a natural property but is also a public good. Hence, human presence in national parks is inevitable.

Recently, there has been an intensive increase in the number of visitors to European national parks. Above-average fauna, flora, and natural resources were found in small areas attract tourists (Schwartz et al. 2012; Millhäusler et al. 2016; Schamel and Job 2017). In addition, tourists are driven by the desire to commune with nature and the pursuit of environmental experiences, but also the need to relax and exercise outdoors(Rogowski 2020; Esfandiar et al. 2021). Tourism often affects the creation of new protected areas. On the other hand, immoderate an excessive one may pose a threat to them (Esfandiar et al., 2018; Mateu-Sbert et al., 2013; Peng et al., 2017; Religa & Adach, 2020; Rodríguez-Rodríguez, 2012; Wu et al., 2020). For many years, research has been conducted in Europe and the world on the management of protected areas in the context of reconciling access to nature with adequate nature protection, as well as monitoring the effects of making them available (Monz et al. 2010; Zhou and Edward Grumbine 2011; Weaver and Lawton 2017; Hu et al. 2018a). Pickering et al. (2010), McCool (2006), Marion and Reid (2001), and Eagles (2002) indicate the need for nature conservation along with rational access to it (Marion and Reid 2001; Eagles 2002; McCool 2006; Pickering et al. 2010). It is also essential for the proper development of the national park area, an extensive network of tourist routes and the availability of shelters located in strategic places, which consequently affects the dynamic increase in visits (Bancheva-Preslavska and Bezlova 2020).

In Poland, the presence of humans for tourist purposes in national parks is strictly defined by the principles of making the park accessible under the Nature Conservation Act (Dz.U. 2004 Nr 92, Poz. 880). Accessibility is defined as enabling people to use the resources of a national park for scientific, educational, recreational, sports, filming, photographing and also for-profit purposes. These vast opportunities brought by making the land available to national parks contribute to the development of tourism both in the park itself and its entire surroundings (Partyka 2010a; Prędki and Park 2017).

In the world, including Poland, exploring little-known places combined with active recreation is becoming a new lifestyle trend. One of the manifestations of this activity is the dynamic increase in the number of visits to national parks in recent years. In 2016, Schagner et al. (2016) conducted a study in which they determined that 205 European national parks are visited by a total of 2 billion tourists a year (Schägner et al. 2016); now, this number is increasing (Stemberk et al. 2018; Religa and Adach 2020; Seebunruang et al. 2022).

We have 23 national parks in Poland, 9 of which are mountainous. About 13 million tourists visit all parks annually. Mountain parks are under pressure as around 8 million tourists visit each year. In most of these areas, tourism in Poland has a seasonal form, where mountain national parks are visited even 75% more often during the peak season than in the off-season (Borzyszkowski 2014). The enormous popularity of mountain parks is primarily due to their natural and landscape attractiveness. According to the researchers, an essential factor in the growth of tourist traffic in a given area is the large availability of tourist facilities of a different nature and standard in its surroundings (Partyka 2010b; Prędki and Park 2017; Bera and Zaremba 2021). It consists of transport accessibility (developed network of roads, railway and air connections), accommodation and catering facilities, and cultural and recreational facilities. One should also not forget about the role of tourist facilities in the area of ​​the park itself, especially the network of trails and shelters. According to Li et al. (2022a, b, c), tourist attractions are the local history of the region, beliefs, traditions, and culture, referred to as cultural tourism. It motivates tourists to discover and experience cultural attractions at tourist destinations. Mountain national parks in Poland are surrounded by towns with great potential for cultural heritage (Mamirkulova et al. 2022). Both in Poland and the world, the development of tourism, in addition to measurable economic, social, and economic benefits, also harm the environment of naturally valuable areas (Stubelj Ars and Bohanec 2010; Weaver and Lawton 2017; Canteiro et al. 2018; Religa and Adach 2020). Limited tourist capacity of parks and the lack of adequate capacity of tourist routes in many places cause devastation of the naturally valuable area. The interaction between tourism and the environment determines the development of tourism. Tourism has a continuous impact on the environment in which it operates, changing it in a favorable direction by rational access to it and a negative one by destroying natural resources (Kiryluk and Borkowska—Niszczota 2009). In 2019, an epidemic broke out in China and quickly became a global pandemic reaching Poland. The pandemic has slowed down the rapidly growing tourism industry in the world and Poland. The health crisis related to the emergence of the COVID-19 virus has affected all critical sectors of the economy, including the tourism industry(Fu et al. 2021). Tourism during the pandemic was temporarily halted. During the COVID-19 pandemic in Poland, national parks, as institutions subject to national regulations, had to close protected areas for tourist traffic (Zenker et al. 2021; Al Halbusi et al. 2022; Li et al. 2022c; Matsuura and Saito 2022). Appropriate management and collective responsibility of society for the natural environment is crucial for adequately functioning the system in national parks and nearby tourist destinations.

The research aimed to analyze the intensity of tourist traffic and tourist capacity in mountain national parks in Poland. Based on the data provided by national parks, an analysis of the effects of anthropopressure resulting from the provision of tourism to these areas was carried out, and the quantitative and qualitative characteristics of the tourism development of mountain national parks and their surroundings were presented. The results of the analysis were used to develop a map of the tourist load of mountain national parks in Poland.

## Methods

### Study area

**Fig. 1:**
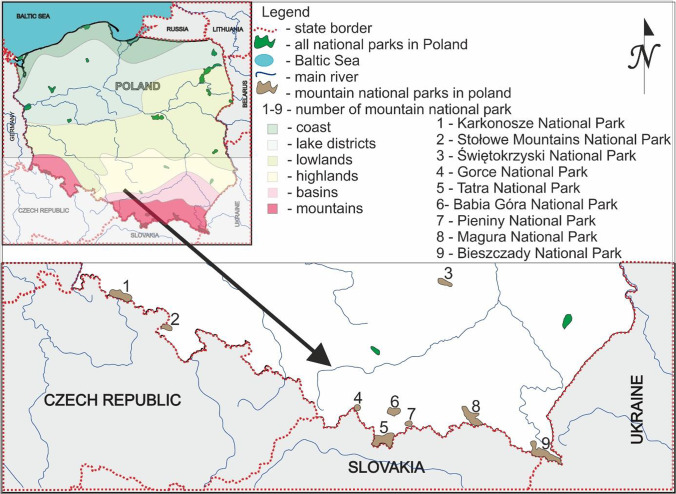
Mountain national parks in Poland, a fragment of the map of Poland

Poland is located in the central-eastern part of Europe and is one of the few European countries with a large variety of environments (access to the sea and mountains). In order to preserve biodiversity, resources, creations and components of inanimate nature as well as landscape values, restore the proper state of resources and natural components, and restore distorted natural habitats, twenty-three national parks were established (Fig. 1).

Due to the diversity of all national parks in Poland, the criteria for selecting a national park as a research area were: the mountain nature of the park and the International Union for Conservation of Nature (IUCN) classification (category II or a protection regime corresponding to this category). Nine national parks were selected for the study, two located in the Sudetes mountain range, six in the Carpathian mountain range, and one in the highlands (Table 1).

**Table 1:** Characteristics of mountain national parks in Poland^a^

Lp	National park	Geographic location	Year of foundation	Area [km^2^]	Highest mountain	
					Name	Altitude above sea level
1	Karkonosze National Park	Sudety mountain range	1959	59.51	Śnieżka	1602
2	Stołowe Mountains National Park	Sudety mountain range	1993	63.49	Szczeliniec Wielki	919
3	Świętokrzyski National Park	Highland range	1950	76.26	Łysica	612
4	Gorce National Park	Carpathians mountain range	1981	70.29	Jaworzyna Kamienicka	1288
5	Tatra National Park	Carpathians mountain range	1955	211.97	Rysy	2499
6	Babia Góra National Park	Sudety and Carpathians mountain range	1954	33.99	Babia Góra	1725
7	Pieniny National Park	Carpathians mountain range	1932	23.72	Trzy Korony	982
8	Magura National Park	Carpathians mountain range	1995	194.38	Wątkowa	846
9	Bieszczady National Park	Carpathians mountain range	1973	292.02	Tarnica	1346

^a^Based on the Central Statistical Office and data provided by employees of national parks.

An interview was conducted, the scope of which included three thematic groups: (1) tourist load of the park, the intensity and spatial nature of tourist traffic in the park (2) tourist management of the park and its surroundings; and (3) the impact of tourist traffic on the natural environment. Additionally, the studies of statistical yearbooks of the Central Statistical Office and data available on the websites of selected mountain national parks were used.

### Methodology

Each year, mountain national parks in Poland are visited by several million tourists, and their number is systematically growing. The number of tourists visiting national parks is monitored through the sale of admission tickets to the area of ​​most tourist routes. In addition, tourist traffic in national parks is controlled by park employees who prepare appropriate additional estimates supplementing entries to the park area at points without tickets and after their closure. Based on the data provided by the employees of the administration of mountain national parks, their website and Central Statistics Office determined the long-term trends in tourist traffic and parameters for the tourist use of the national park (tourist traffic density, tourist trail density) and tourist traffic intensity.

The density of tourist traffic in the national park is determined using the formula:

$${d}_{t}=\frac{N}{P}$$

where:

*d*_t_: tourist density [(person × year) / km^2^]
*N*: the average annual number of tourists visiting the national park [person × year]
*P*: area of ​​the national park [km^2^]

The density of tourist routes is determined based on the formula:

$${d}_{r}=\frac{l}{P}$$

where:

*d*_r_: density of tourist routes [km / km^2^].
*l*: total length of hiking trails in the national park [km].
*P*: area of ​​the national park [km^2^].

The intensity of tourist traffic is determined based on the formula:

$${I}_{tt}=\frac{N}{l}$$

where:

I_tt_: Tourist traffic intensity [(person × year) / km].
N: the average annual number of tourists visiting the national park [person × year].
l: total length of hiking trails in the national park [km].

## Results

### The intensity and spatial character of tourist traffic in mountain national parks

**Fig. 2:**
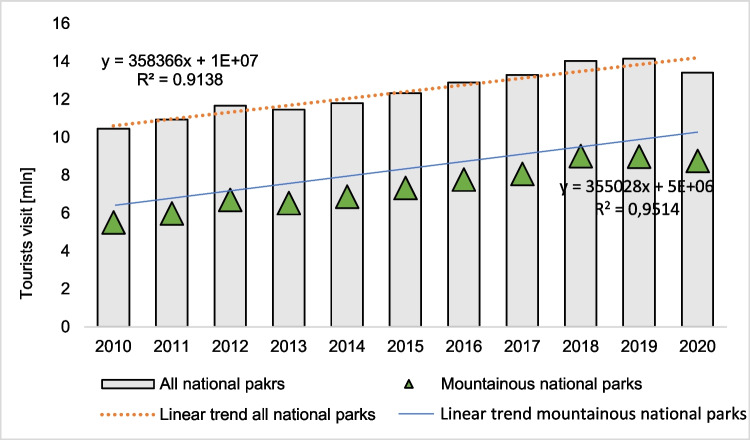
Tourist visits to Polish national parks in 2010–2020

National parks in Poland are prevalent among visitors and are places of concentration for tourists (Wilgat 2002; Kruczek and Przybyło-Kisielewska 2019). The number of people visiting parks increases yearly (Fig. 2). In 2010, Polish national parks were visited by nearly 10.5 million tourists, in 2016 almost 13 million, and in 2019 over 14 million. In 2020, a decrease in tourist visits was observed, which was caused by the COVID-19 pandemic, which began at the beginning of the year in the world and Poland (Abbas et al. 2021; Volgger et al. 2021; Matsuura and Saito 2022). The COVID-19 pandemic has hit almost every region in the world. The crisis has affected many countries and areas of the world, state borders have been closed, and travel restrictions have been introduced (Wang et al. 2021; Li et al. 2022a). In Poland and Europe, most national parks have been closed to tourists, or changes have been introduced to limit the number of tourists allowed to stay in national parks (Niezgoda and Markiewicz 2021; Škare et al. 2021). Travel and tourism have been found to promote the spread of the highly infectious COVID-19 virus (Lakshmi Singh et al. 2021; Zenker et al. 2021; Shin et al. 2022). Nevertheless, when analyzing the tourist traffic in the area of ​​national parks in Poland over the last 10 years, it can be noticed that its level indicates a continuing upward trend (Fig. 2). This increase is, on average, 358 thousand tourists per year (high correlation *R*^2^ = 0.9138). A similar trend occurs in the case of changes in tourism in mountain national parks. There is an upward trend in annual cycles by approximately 355 thousand more tourists. The level of correlation for the obtained dependence is very high and amounts to *R*^2^ = 0.9514. Considering the number of tourist visits to all national parks in Poland and comparing it only with those of mountain nature, a large diversification of the intensity of tourist traffic is noticeable. Among the entire group of tourists visiting national parks in Poland, nearly 60% are visitors to mountain national parks (Central Statistical Office 2021). Above-average landscape values cause it, and a unique natural environment occurs only in the area of ​​mountain parks. A characteristic feature of such areas is the availability of hiking trails of varying difficulty for families with children and demanding, experienced tourists (Hibner, 2013). Due to these factors, the number of tourist visits to mountain parks is systematically increasing from year to year. In 2010, mountain national parks were visited by 5.51 million tourists; in 2015, it was already 7.32 million tourists and in 2020, 7.75 million tourists, which is 53%, 59%, and 65% of all entries, respectively, to all national parks in Poland.

**Fig. 3:**
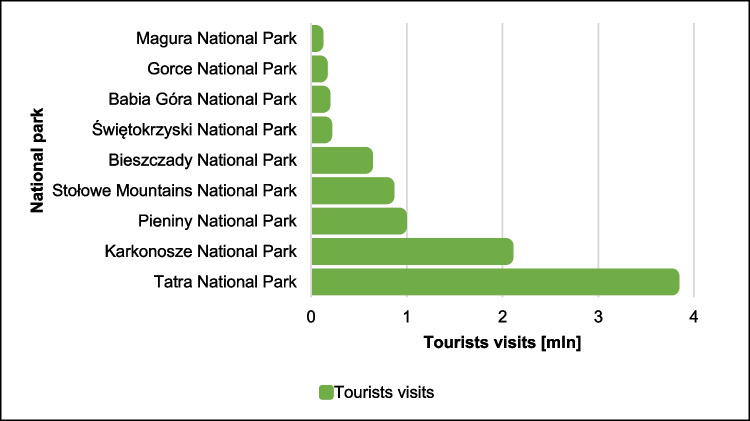
Tourist traffic in mountain national parks in Poland

Equally characteristic is the immense diversification of tourist traffic in individual mountain national parks (Partyka 2010a). In nine Polish mountain national parks, the turnout of visitors varies greatly. The Tatra National Park and the Karkonosze National Park deserve special attention. They are the most visited national parks in Poland (Fig. 3). The highest attendance is observed in the Tatra National Park—about 3.8 million tourists a year. Not much less, more than 2 million tourists visit the Karkonosze National Park every year. Tourist visits to these two national parks account for as much as 43% of tourist entries to all national parks in Poland and 71% of tourist entries to mountain national parks.

In four mountain national parks (Magura, Świętokrzyski, Gorce, Babia Góra), the annual number of tourists does not exceed 150,000, which is about 5% of entries to mountain national parks. About 0.7 million people visit the Bieszczady National Park, the Pieniny National Park, and the Stołowe Mountains National Park by about 1 million. There is a specific dependence on the above-mentioned national parks. These are relatively young mountain national parks, and tourists visit them less often than those established earlier. The reason for this is probably the lack of recognition of the protected area among tourists and the simple ignorance of the existence of public good and its attractiveness. In addition to the popularity of the area, an additional factor that determines the number of tourist visits is its surroundings (availability of holiday resorts, catering facilities) and possible access by public transport.

**Fig. 4:**
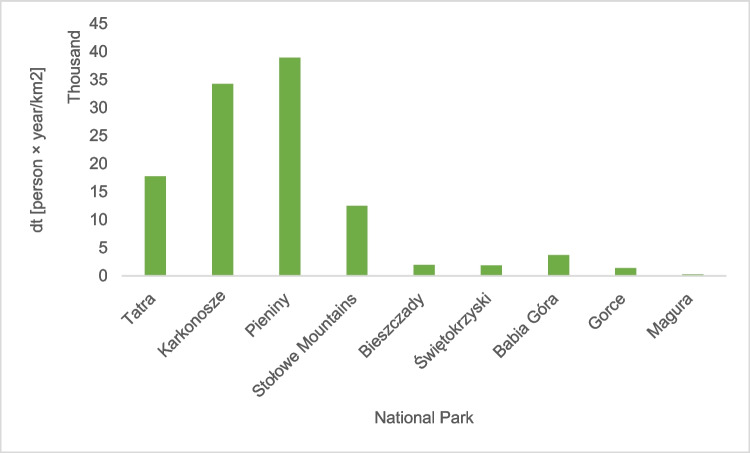
Tourist traffic density

The notion of tourist density is closely related to the volume of tourist traffic and its intensity. The interest of tourists shows the number of visits to a given area. In this respect, the highest density was observed in the Pieniny National Park (38,937 people per 1 km^2^), the Karkonosze National Park (34,264 people per 1 km^2^), the Tatra National Park (17,785 people per 1 km^2^), and the Stołowe Mountains National Park (12,509 people per 1 km^2^) (Fig. 4). These are the most stunning mountain national parks. In other parks, the tourist traffic density does not exceed 4 thousand people on 1 km^2^.

### Tourist development of mountain national parks

The first important aspect in defining the spatial development of mountain national parks is the surrounding, which are closely related to the availability of accommodation and catering facilities and the transport connection (network of roads, highways, and distance from rail and air transport). The second parameter determining the tourism development of mountain national parks is the park’s attractiveness as a protected area, its tradition, natural values ​​and recognition.

The results indicate an essential role of factors determining the tourist attractiveness of mountain areas of national parks in Poland, about which numerous authors write (Parzych & Zienkiewicz, 2012). Kruczek and Przybyło-Kisielewska (2019) and Liszewski (2009) believe that the tourist attractiveness and popularity of parks among tourists are mainly influenced by recreational values ​​(hotel and catering facilities) and extraordinary landscape. According to other researchers, communication accessibility and a well-developed road network, which makes it easier to reach the national park, are very important (Parzych and Zienkiewicz 2012). Most mountain national parks are well-connected, but the quality of access roads is poor, which means that access to them takes much time, which translates into the number of tourist visits (Myga-Piątek and Jankowski 2009). A completely different view on the quality of communication and its impact on the environment is presented by Aman et al. (2022). According to them, in the world, in Asia (China–Pakistan), the excellent condition of roads and improvement of infrastructure has a positive effect on the improvement of the quality of life of the inhabitants of rural areas and Local communities. Good communication facilitates movement, opening the market to new opportunities and reducing poverty. However, they do not notice any beneficial effects on the natural environment. Development of infrastructure often causes environmental devastation (Mamirkulova et al. 2020) indicate that the development of the tourist infrastructure of the New Silk Road has a positive effect on the inhabitants’ quality of life through the sustainable development of tourism in Kazakhstan but negative on the natural areas. The authors point out the activities increasing the standard of living of the region’s inhabitants and the competitiveness of tourist destinations.

**Fig. 5:**
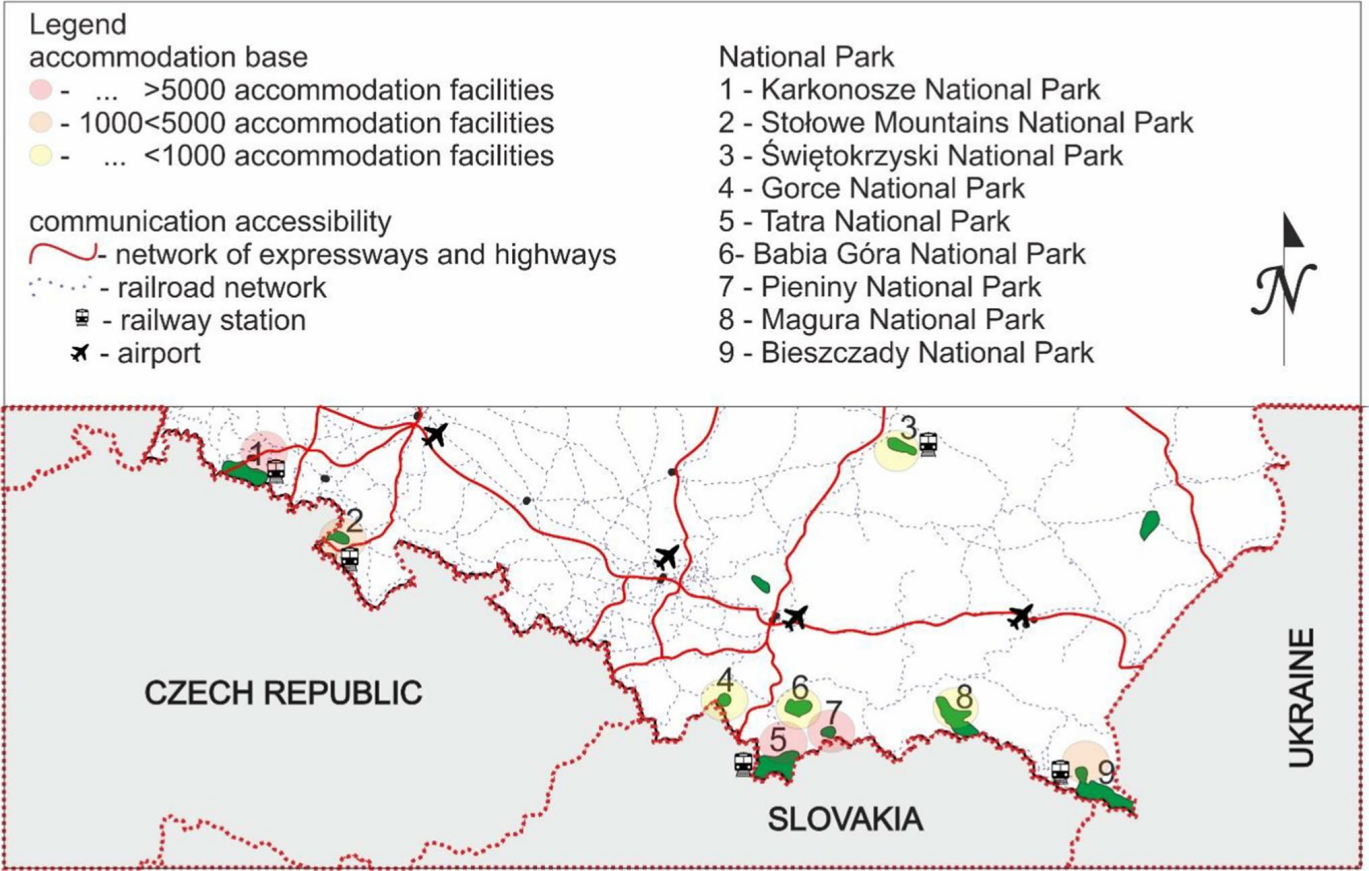
A fragment of the map of Poland showing the level of tourism development and transport accessibility of the surroundings of mountain national parks

It was noticed that Polish mountain national parks, which tourists more often visit, have a better-developed accommodation base in the form of hotels, boarding houses, and private holiday homes, which is consistent with the research by Parzych and Zienkiewicz (2012). National parks such as Karkonosze, Pieniny, and Tatra are the most frequent destination in the mountains. The quantitative analysis of the accommodation base and the availability of expressways (motorways, expressways) as well as the availability of a railway connection determined in the research have shown that only two national parks have expressways nearby (Karkonosze, Stołowe Mountains), and the best connected by rail are Karkonosze, Stołowe Mountains, Tatra, Świętokrzyski, and the Bieszczady National Park. The research also showed that the location of airports does not significantly impact the intensity of tourist traffic in mountain national parks. In the light of the presented results, many similarities in tourism development and transport accessibility in the analyzed parks are noticeable (Fig. 5). The natural values ​​of parks and their tradition have the most significant impact on tourist traffic. Natural values ​​and attractiveness of protected areas seem to be the greatest asset of national parks, causing considerable disproportions in tourist traffic. The parks attract the most significant interest, where the topography is diverse and the landscape is unique. The areas of mountain national parks are the more frequently visited trails, and the areas available in the park are more difficult to overcome. Scientific research by Li et al. (2022a, b) also indicates a prominent role of cultural tourism, which significantly impacts economic development and is a pillar of the tourism industry. Tourists often choose a naturally valuable area with a fascinating history and tradition as their destination for a journey. Considering all the factors mentioned above about tourist attractiveness are fulfilled for the Tatra National Park and the Karkonosze National Park, which translates into many tourists in these parks (Fig. 3).

Very well-developed tourist infrastructure is closely related to tourist visits, which includes, in particular, a network of routes and shelters (Table 2). Shelters provide accommodation and catering facilities in the areas of national parks. They are both a destination and a stop for tourists to rest before continuing their journey. Twenty-five tourist shelters operate in mountain national parks in Poland (Table 2). Most of them, ten shelters, are located in the Karkonosze National Park, while eight operate in the Tatra National Park. There are two shelters in the Stołowe Mountains National Park and the Świętokrzyski National Park, three shelters in the Bieszczady National Park, and one shelter in the Babia Góra National Park. In the Gorce, Magura, and Pieniny National Parks, there are no them. The mass form of tourism makes hostels a base for tourist traffic. In mountainous national parks, especially those with large denivelations, they are viewpoints and a marina where tourists have the opportunity to rest before continuing their hike on more demanding routes. In most cases, the routes leading to the shelters are adapted even for people with physical disabilities or the elderly.

**Table 2:** Quantitative and qualitative characteristics of hiking trails in mountain national parks in Poland

National Park	Shelters^c^	Tourist routes				
		Tourist hiking trail	Tourist bicycle trail	Tourist canoe trail	Tourist horse trail	Ski slopes ^c^
Babia Góra National Park	1	49.4	2	-	2	6 (6,8^b^)
Bieszczady National Park	3	467	51	-	35	-
Gorce National Park	-	125	58	-	60	0.3 (125^a^)
Stołowe Mountain National Park	2	109	143,5	-		32
Karkonosze National Park	10	136		-		14,7
Magura National Park	-	145.5	110	-	16	-
Pieniny National Park	-	35.2	13	River Dunajec		(35^a^)
Świętokrzyski National Park	2	33.1	34		9	-
Tatra National Park	8	275	37.6	-	-	24,8

^a^Ski tourism can be practiced on all routes available for hiking.

^b^Data made available on the website of the park.

^c^Central Statistical Office based on data from the Ministry of Environment.

Various types of trails are marked out in Polish national parks. These include hiking, biking, canoeing, and horse and skiing routes. It is worth emphasizing that tourists in national parks can, in principle, only walk on marked hiking trails (Pickering et al. 2010; Imran et al. 2014; Prędki and Park 2017; Kołodziejczyk 2019). When creating hiking trails in the mountain national parks, the landscape and cognitive values ​​of a specific area are essential, while, due to the rugged terrain and numerous natural habitats, tourist routes in mountain national parks in Poland are created with safety for tourists and environmental protection (Stasiak et al., 2014).

In mountain national parks in Poland, there are the most hiking trails, the average length of which is approximately 153 km, and the total length for all-mountain national parks is 1375 km. The most extensive network of routes is located in the Bieszczady National Park, where tourists have 467 km of hiking trails and roads at their disposal. The shortest network of routes, only 33.1 km, has the Świętokrzyski National Park (Fig. 3).

Eight out of nine mountain national parks have marked bicycle trails. There are no such routes in the Karkonosze National Park. The most significant number of bicycle routes with a length of 143.5 km and 110 km were marked out in the Stołowe Mountains National Park and the Magura National Park, respectively. The shortest length of the marked-out bicycle route is in the Babia Góra National Park, 2 km. The total length of bicycle routes is 449.1 km, similar to the length of all hiking routes in the Bieszczady National Park.

The tourist canoe trail is marked only in the Pieniny National Park—canoeing trips the Dunajec River.

Horse paths have been marked out in five of the nine mountain national parks. Their total length is 122 km. The most significant number of hiking trails is in the Gorce National Park (60 km), and the least in the Babia Góra National Park (2 km). There are no such routes in the Karkonosze, Stołowe Mountains, Pieniny, and Tatra National Park.

The last type of hiking trail is the pistes. Their presence is closely related to winter sports and depends mainly on the park’s location and weather conditions. Currently, the slopes have been marked out in six mountain national parks—Babia Góra, Gorce, Stołowe Mountains, Karkonosze, Pieniny, and the Tatra National Park. The length of the slopes varies and ranges from 0.3 to 32 km. In the Gorce and Pieniny parks, ski tourism can be practiced on all routes available for hiking.

Another element that characterizes national parks is the density of tourist routes. This parameter was determined using the information on the area of ​​the mountain national parks and the length of hiking trails available to tourists.

**Fig. 6:**
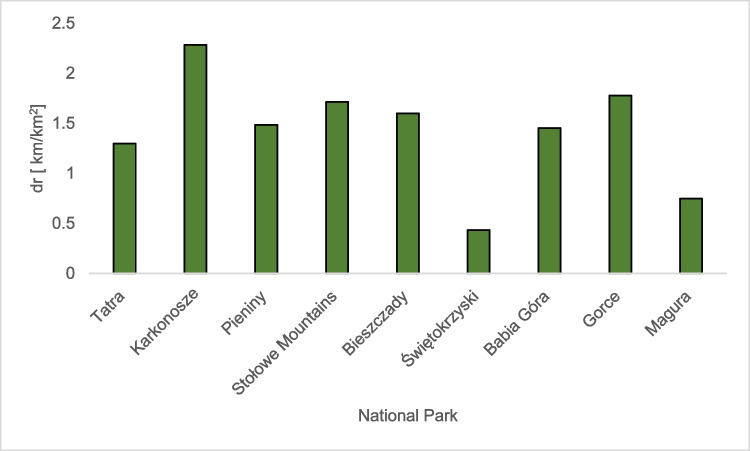
The density of hiking trails in mountain national parks

The average density of hiking trails in mountain national parks is 1.42 km/km^2^. The Karkonosze National Park (2.29 km/km^2^) has the highest density of tourist routes, while the Świętokrzyski National Park (0.43 km/km^2^) is the lowest (Fig. 6).

Setting out tourist routes is a long and labor-intensive process. Designating tourist routes is designed to adapt and provide tourists with interesting tourist values and protect the most valuable, incredibly natural values. An adequately educated tourist infrastructure should ensure safety (for the environment and tourists), communicate many places with each other (an extensive network of routes) and constitute observation points for natural and cultural values without disturbing the natural environment. It causes significant differences in the density of hiking trails. On the other hand, when determining the density of hiking trails, one should mention the intensity of tourist traffic on the trails. Both of these parameters are closely related and characterize the tourist development of the park area (Kruczek and Przybyło-Kisielewska 2019).

**Fig.7:**
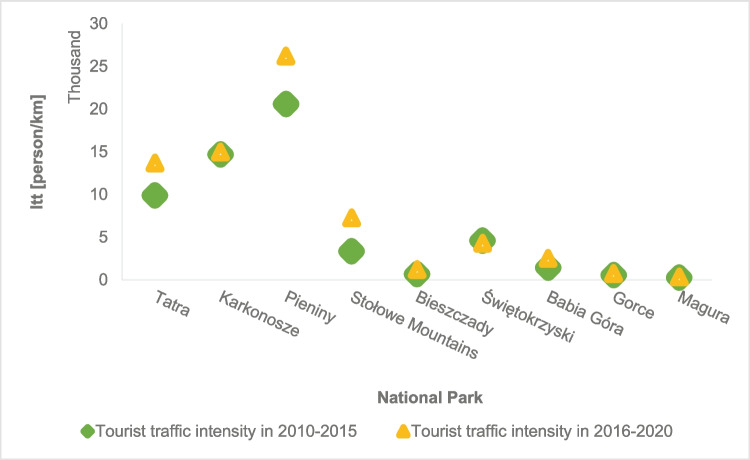
The tourist traffic load in mountain national parks

Another parameter characterizing parks is the tourist traffic load defined by the intensity of tourist traffic. The highest intensity of tourist traffic characterizes Pieniny, Karkonosze, and Tatra National Park. The tourist traffic load in these parks in 2010–2015 amounted to an average of almost 16,000 persons/km, and already in 2016–2020, it increased to over 18,000 persons/km. A disadvantageous feature is the lack of space in these parks to expand the route network. The continuing upward trend in the number of tourists visiting national parks (Fig. 2) allows us to assume that the tourist capacity of these areas will soon be exhausted. In the remaining mountain national parks, the intensity of tourist traffic is about 5 thousand people on a kilometer of a hiking trail (Fig. 7).

### Protection zones in national parks

**Fig. 8:**
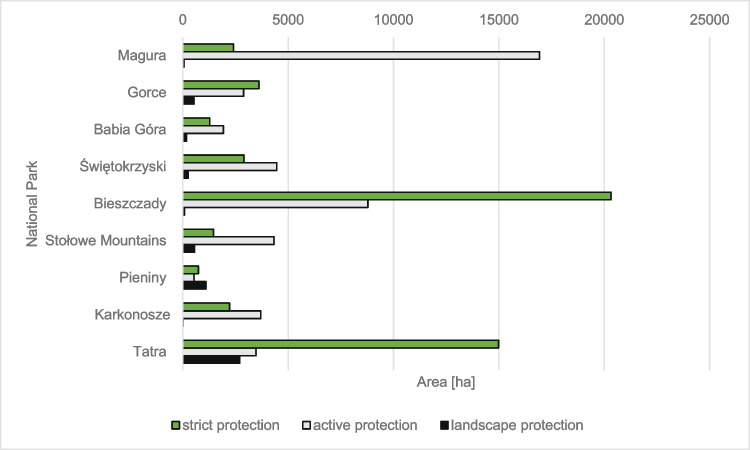
Mountain national parks by protection category

There are three different protection zones in Polish national parks: a zone of strict, partial and landscape protection (Fig. 8). The strict protection zone is based on the complete preservation of ecosystems and all processes occurring in them in a natural state, e.g., natural regeneration, decomposition of lying wood. The areas of the park under strict protection are made available only for scientific purposes. In the partial (active) protection zone, measures are taken to restore the proper species composition and appropriate structure. The third zone is the landscape protection zone, where it is possible to use the areas sustainably for people, e.g., forests and glades. However, it is vital to keep all-natural values (Religa et al. 2021).

Taking into account the mountain national parks in the Bieszczady and Tatra National Parks, the strict protection zone covers about 70% of the park area. The smallest area of ​​the strict zone is in the Magura National Park, which covers only about 10% of the park’s area. The active protection zone in mountain national parks ranges from 20 to 87%. The smallest area of ​​the active protection zone is located in the Tatra National Park, accounting for approximately 20% of the entire park area, and the largest is in the Magura National Park and accounts for approximately 87%. The landscape protection zone covers small areas and usually constitutes about 1–15% of the park area. However, compared to all mountain national parks, the Pieniny National Park stands out significantly—the landscape protection zone covers about 46% of the park area. The smallest area of ​​landscape protection is in the Bieszczady and Karkonosze National Parks; it accounts for about 1% of the parks’ area.

## Discussion

### Influence of tourist traffic on the natural environment of mountain national parks

Tourism is a crucial element of the functioning of national parks, and its development brings benefits (local openness, new jobs, dissemination of an exciting region) and adverse effects. All human activities, both planned and accidental, affect the natural environment and are called anthropopressure (Almeida Cunha 2010; Koźmiński and Michalska 2016). Its negative consequences for protected areas, in particular mountainous areas, are as follows: (1) activities affecting the trampling of trails and the related destruction of vegetation and soil erosion, (2) disturbing the peace of animals and their synanthropization, and (3) waste left on the trails or in their vicinity (Hu et al., 2018a, b; J. C. Kuniyal et al., 1998; Jagdish C. Kuniyal, 2005; Religa & Adach, 2020, 2021b).

### Anthropopression

**Fig. 9:**
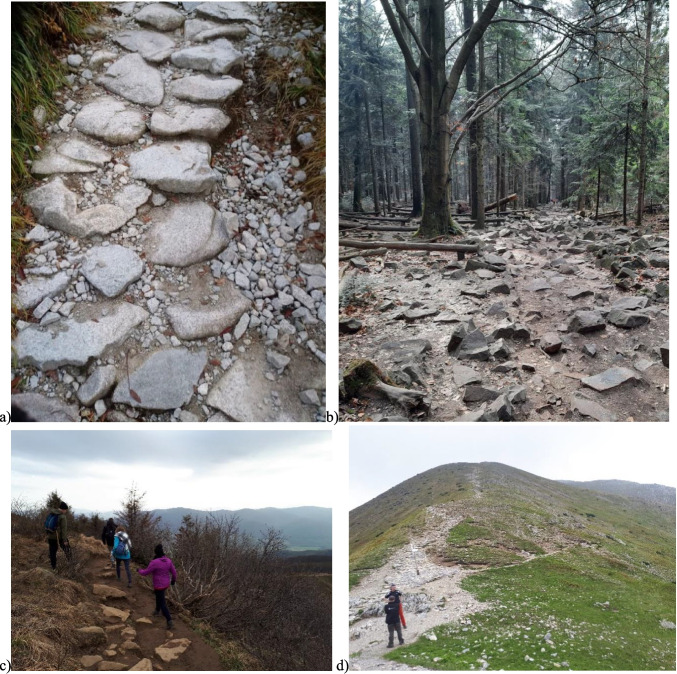
Destruction of tourist routes: **a**) Pieniny National Park, **b**) Świętokrzyski National Park, **c**) Bieszczady National Park, and **d**) Tatra National Park

In Poland, the most outstanding tourist activity on tourist routes is observed at the peak of the tourist season (May–September). During this period, the routes are visited daily by up to 35,000 tourists (Religa and Adach 2020; Religa et al. 2021). During the seasons in European parks, there are an average of 4000 visitors to the trails per day on tourist routes, and at the peak moment, this number can even be around 15,000 tourists (Millhäusler et al. 2016; Barros et al. 2019). On routes that do not have adequate capacity, intensive tourism causes devastation of routes and vegetation, causing significant transformations in the immediate vicinity and destroying the natural or artificial cover of tourist routes. The routes are widened by trampling them and creating new tourist paths. The nearby vegetation is often destroyed (Fig. 9).

### Animal synanthropization

**Fig. 10:**
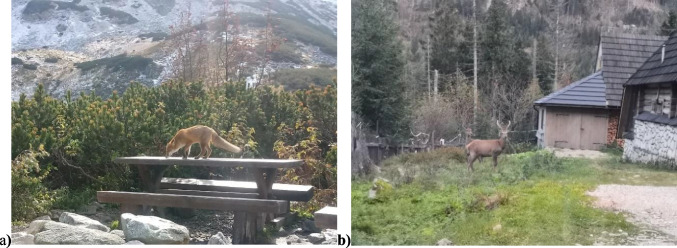
Animal synanthropization: **a**) a fox looking for food among people and **b**) a doe next to farm buildings

Animals living in the area of ​​the trails are often scared away by excessive noise, which is observed in all parks (Iglesias Merchan et al. 2014). Tourism arouses anxiety for animals and over time causes them to assimilate, which often supersedes their natural instincts (Fig. 10). Instead of getting food, animals start to get them from tourists, which disturbs the natural order of the natural cycle.

### Waste left by tourists in national parks

**Fig. 11:**
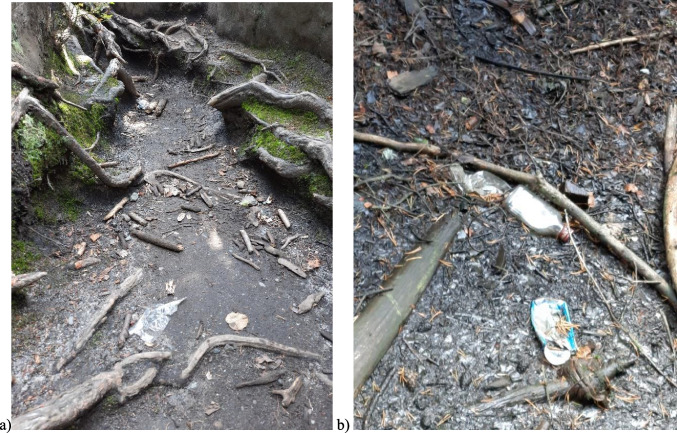
Waste on tourist routes: **a**) Stołowe Mountains National Park and **b**) Karkonosze National Park

A fundamental problem for the environment of protected areas is the waste left by tourists in inappropriate places (Hu et al., 2018a, b; J. C. Kuniyal et al., 1998; Religa and Adach, 2020; Rodríguez-Rodríguez, 2012) (Fig. 11). Tourists do not always follow the rules of national parks, which often results in contamination of these areas (Wu et al. 2020). In the area of ​​national parks in Poland, there are usually no waste collection sites, and if they are, they are located in the areas of tourist infrastructure. Visitors to national parks are required to take the waste with them. Waste left on and in the vicinity of the routes reduces the natural value of a given area, destroys the natural landscape, and negatively affects the fauna and flora (Ghazvini et al., 2020; Mateer et al., 2020; Rogowski, 2020; Schamel and Job, 2017; Schwartz et al., 2012). Waste left on the trails attract wildlife animal to the hiking trails (Rodríguez-Rodríguez, 2012). Some types of waste, especially plastic, glass, and metal packaging, can also be a death trap for animals (Kolenda et al. 2015).

### International ideas supporting protected areas

The problems resulting from accessing tourists mountain national parks reflect the awareness of the community visiting the parks. The conducted research proves that such factors as education, age, ecological awareness, the social class represented by tourists, and educational and information activities carried out by national parks significantly impact the behavior of tourists in the natural environment. International initiatives educate the public about ecology and support the natural environment. The most popular waste-related initiatives in national parks are the “Zero Litter Initiative,” “Leave No Trace,” and “Plogging.”

The Zero Litter Initiative (ZLI) is based on removing waste from hiking and climbing routes by tourists visiting an area of ​​natural value. It is an action promoting appropriate tourist behavior, and its success depends on the enthusiasm and willingness of tourists to join the initiative. Huangshan National Park first proposed this initiative in 2013 with incentives to participate such as bottled water, postcards, and other souvenirs. The entire ZLI project was aimed at improving the ecological awareness of tourists and publicizing the problem of waste in the mountain national parks, where its removal is complicated (Hu et al., 2018a, b).

The idea of ​​ “Leave No Trace” (LNT) originated in the 1990s of the twentieth century, initially having a local reach. It spread quickly and began to be widely used in protected areas in the USA. Its main goal is to raise visitors’ awareness about the potential adverse effects associated with their visits and educate visitors about the appropriate preparation for expeditions to areas of natural value to avoid or minimize the impact of human presence on the natural environment. Currently, the educational program consists of 7 basic rules that must be followed. They refer to good preparation for expeditions and emphasize not leaving behind traces that may harm the natural environment (Marion and Reid 2001).

Plogging is a trend that originated in Sweden. Its name refers directly to the initiative and is a combination of the words: Swedish opp, which means to pick up, gather, and jogging, which means to run. The initiative has recently been implemented. The first such slogan was introduced in 2016. It is based on a selfless collection of waste during outdoor exercise. Plogging is not only about training but also taking care of the environment.

Interestingly, such an initiative is gaining more and more interest. It is an activity not only in big cities, mountains, forests or beaches. It also affects protected areas such as national parks. In Poland, a series of educational films have been created in the Tatra National Park, in which the topic of the plogging initiative is also discussed as an action with a positive impact on the natural environment and shaping appropriate attitudes among society (Religa and Adach 2021; Tatrzański Park Narodowy 2022).

Nowadays, such actions play a vital role, and their wide publicity draws attention to the problems of the natural environment. This is especially true of areas that are intensively used for tourism.

### Tourist burden of mountain national parks in Poland

**Fig. 12:**
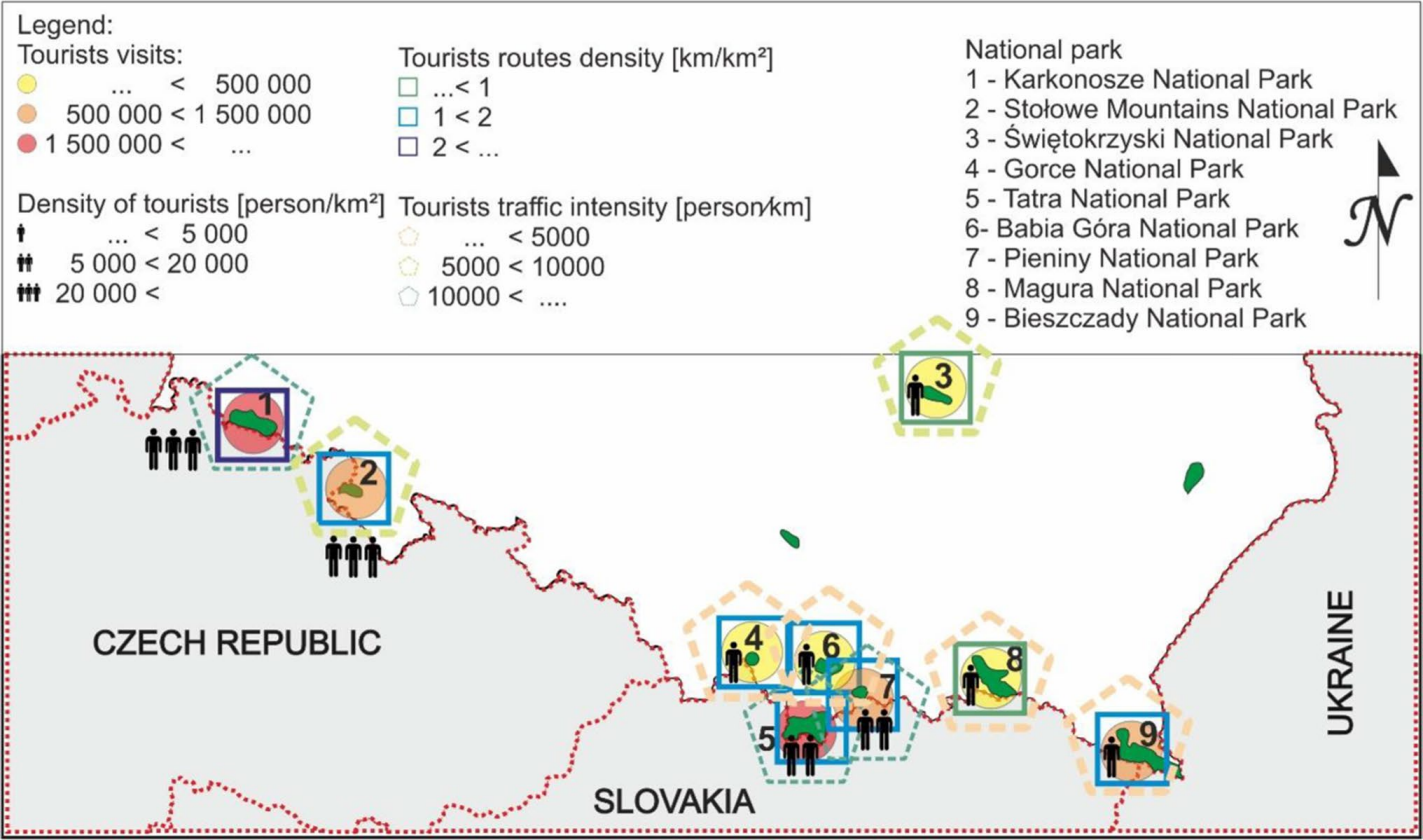
A fragment of the map of Poland with the location of 9 mountain national parks and the tourist burden on national parks

The tourist burden is closely related to the development of the area of ​​the mountain national park, the attractiveness of its area (network of tourist routes, catering and accommodation facilities inside the park in the form of shelters), tradition, and the level of development of the surroundings (transport accessibility: the network of expressways and highways and railway connections). In mountain national parks in Poland, the average density of trails is 1.42 km/km^2^, the highest density is in the Karkonosze National Park, and the lowest in the Świętokrzyski National Park. Nevertheless, taking into account the tourist traffic density in the last 5 years, the highest has been observed in the Pieniny National Park, around 40,000 people per 1 km^2^ of the park area. A parameter closely related to the density of routes and tourist traffic density is the tourist traffic intensity parameter. It was found that the highest intensity of tourist traffic is observed in the Pieniny, Karkonosze, and Tatra National Parks (Fig. 12).

Notably, a large number of tourists, highly concentrated on marked tourist routes, pose a threat to the natural environment. Too intense tourist penetration hardly reverses changes in the natural environment of protected areas.

### Policy recommendations

All national parks in Poland conduct volunteer campaigns. Pro-ecological activities related to voluntary actions concerning the natural environment manifest the social responsibility of people visiting national parks in Poland and around the world. Corporate Social Responsibility (CSR) covers aspects of the internal environment and relations with stakeholders in the social context (Graja-Zwolińska and Maćkowiak 2016). By definition, it is corporate social responsibility, which means the responsibility of an organization for the impact of its decisions and actions on society and the environment (Graja-Zwolińska and Maćkowiak 2016; Fu et al. 2021). The concept of CSR is a strategy covering all aspects of enterprises, thanks to which organizations have the opportunity to implement good practices in every dimension, regardless of the industry. National parks, as institutions, have the task of working out a balance between conservation activities and the implementation of economic and social undertakings. Tourism is necessary for the proper operation of national parks, which is the driving force of the business. On the other hand, the national parks must be responsibly managed by persons supervising and performing management functions in the national parks in a manner consistent with the prevailing rules. Tourism in national parks is an industry that is constantly developing, and its dynamic growth means that some areas of natural value do not have adequate capacity. In the vicinity of mountain national parks in Poland, there are towns with a long tradition, beautiful for tourists due to their history, with well-developed facilities in the form of adequately prepared accommodation, service, and catering facilities. According to Emphandhu and Polpiwat (2006), community-based tourism should include local involvement—action from national parks, particularly in creating new jobs. It can arise when the local population realizes the benefits of tourism in national parks.

The local community’s willingness to engage in tourism also seems to be an essential aspect. The rapidly growing tourism industry in the world and Poland was halted by the emergence of an epidemic, which quickly turned into a pandemic. The health crisis related to the COVID-19 virus has affected all critical sectors of the economy and highlights the problems associated with crisis management (Geng et al. 2022; Yu et al. 2022; Zhou et al. 2022). During the COVID-19 pandemic in Poland, national parks as institutions had to adapt their strategies to the ongoing crisis, which prohibited tourists from entering their area. In many institutions of national parks in Poland, the use of social media for communication purposes has begun. According to Jaffar et al. (2019 and Abbas (2021), the use of social media by institutions positively affects marketing and allows for better results. When the first wave of COVID-19 subsided in the summer of 2020 and after the restrictions on the closure of protected areas in Poland were lifted, there was a peak of tourist visits to mountain national parks, which caused a significant burden on naturally valuable areas. Undoubtedly, the sudden and large intensity of tourist traffic caused significant degradation of the natural environment, which hibernated for several months. On the other hand, given the economic and economic crisis, reopening protected areas was salvation for failing tourism-related businesses (Geng et al. 2022). According to Zhou et al. (2022), the crisis that hit the world caused by the COVID-19 virus also affected the private spheres of society. The period of shutdown led to changes in people’s attitudes toward life. The recommended social distance was considered the most effective preventive protocol to minimize transmission of the deadly COVID-19 virus (Yu et al. 2022). The coronavirus pandemic influenced human behavior and made society dependent on mobile devices and social media. On the one hand, social media spreads unique places with unique history, value, and natural value. On the other hand, they make everything available online, which makes the desire to learn and discover through experiencing secondary.

Appropriate management and collective responsibility of society for the natural environment is crucial for adequately functioning the system in national parks and nearby tourist destinations.

## Conclusions

Providing tourist access to national parks in the world and Poland is part of their ideas; hence, the presence of a human tourist is inevitable and results from the Nature Conservation Act (Dz.U. 2004 Nr 92, Poz. 880). The dynamic development of tourism opens up new opportunities for the areas of national parks in Poland and the world. On the one hand, these are, among other things, economic benefits that also contribute to local openness. An important aspect is also popularizing national parks and engaging tourists in activities for nature protection, which leads to the creation of new areas under protection. On the other hand, tourism has many negative consequences for the natural environment. The number of people visiting national parks is increasing in Poland and the world. This is due to the appropriate tourism development of the park and its surroundings. The tradition and distribution of the naturally valuable area are also important. Some of the protected areas, especially the relatively small ones with high landscape values, considered attractive and popular places, are subject to high tourist pressure, making the protected area easily degraded. This is especially true of mountain parks, where the terrain is difficult to access. Much depends on people visiting national parks and their attitudes towards the environment. Tourists staying in national parks should move only along designated tourist routes, following their intended use. Unfortunately, too many tourists, highly concentrated on marked hiking trails, endanger the natural environment through inappropriate behavior.

The consequences of inappropriate behavior of tourists are frequent land degradation, soil erosion, and destruction of plant biodiversity. Tourism also causes anxiety among wild animals, making them adapt by displacing their natural instincts over time. Animal synanthropization is a virtually irreversible process; hence, it is perilous. An important problem for the natural environment of mountain national parks is the waste left by tourists in prohibited places. Waste left on the routes and in their vicinity gives the impression of a lack of aesthetics, reduces the natural value of a given area, destroys the natural landscape, and negatively affects the fauna and flora. The waste left on the trails often attracts wild animals, which makes it too close to the tourist routes that tourists can follow. The ease of obtaining waste makes it a food source for animals while disturbing the natural predatory instincts. In addition, waste disturbs the functioning of forest ecosystems, resulting in the vegetation existing in natural conditions dying out. Decaying waste causes the growth of pathogenic bacteria and dangerous fungi. In addition, anthropopression causes an enormous transformation of the routes in their immediate vicinity and the destruction of the cover (natural or artificial) of the routes, contributing to soil erosion. It is the result of excessive tourism. In order to prevent or reduce the impact of tourism on the natural environment, educational activities are carried out to raise the ecological awareness of the community visiting national parks. These include the international campaigns: Leave No Trance, Plogging, and Zero Litter Initiative.

Tourism development is key to the proper functioning of national parks; however, if not properly cultivated, it poses threats to the fauna and flora of such areas. The accessibility to tourism in mountain national parks shows that tourism, despite its consequences, is necessary to maintain sustainable development. Contact with nature is a way of managing people’s free time. The interest in the areas of mountain national parks in Poland shows that the need to protect the natural environment is evident. Developing harmony between tourism and the natural environment is fundamental.

## Declarations

### Data availability

The datasets used or analyzed during the current study are available from the corresponding author on reasonable request.

**Ethical approval**

Not applicable.
